# Associations between active commuting to school, sleep duration, and breakfast consumption in Ecuadorian young people

**DOI:** 10.1186/s12889-019-6434-9

**Published:** 2019-01-18

**Authors:** Emilio Villa-González, Francisco J. Huertas-Delgado, Palma Chillón, Robinson Ramírez-Vélez, Yaira Barranco-Ruiz

**Affiliations:** 1 PROFITH Research Group, Department of Physical Education and Sport, Faculty of Sport Sciences, University of Granada, Granada, Spain; 2 School of Physical Education, National University of Chimborazo, Riobamba, Ecuador; 3 Teaching School La Inmaculada, University of Granada, Granada, Spain; 4 Center of Studies in Physical Activity Measurements, School of Medicine and Health Sciences, Universidad del Rosario, Bogotá, Colombia

**Keywords:** Active commuting, Sleep, Breakfast, Young, Latin-Americans, Public health

## Abstract

**Background:**

Daily behaviours such as active commuting to school (ACS) could be a source of physical activity, contributing to the improvement of youth cardiovascular health, however, the relationship between ACS and other aspects of a youth’s health, such as sleep duration and breakfast consumption, require further clarification. The aims of this study were therefore: 1) to analyse the prevalence of modes of commuting to school, sleep duration, and breakfast consumption by age groups and gender, and 2) to analyse the association between ACS, sleep duration recommendations, and breakfast consumption by age groups and gender.

**Method:**

This cross-sectional study included 732 school-aged students of low-middle socioeconomic status, categorised into children (10-12 yr), young adolescents (13-15 yr), and older adolescents (16-18 yr). Modes of commuting to/from school, sleep duration, and breakfast consumption were self-reported. Logistic regression models were fitted to examine the association between ACS, sleep duration and breakfast consumption, analysed according to age groups and gender.

**Results:**

The percentage of students meeting sleep duration and daily breakfast recommendations was lowest in older adolescents, and highest in children (6.3% versus 50.8% *p* < 0.001, and 62.1%, versus 76.8%, *p* = 0.001, respectively). Young adolescents and girls who met the sleep duration recommendations were more likely to be active commuters than their counterparts (OR = 4.25; 95% CI = 1.81 to 9.92, p = 0.001 and OR = 2.89; 95%CI = 1.01 to 8.27, *p* = 0.04, respectively)*.*

**Conclusion:**

Young adolescents (13-15 yr) and girls who met the sleep duration recommendations during school days displayed a positive association with ACS. There was no association between ACS and breakfast consumption for any of the age groups or gender. Children (10-12 yr) were those that best meet with the adequate sleep duration and breakfast consumption recommendations.

## Background

The prevalence of overweight and obese young people, both from developed and non-developed countries has increased around 47% in the last 10 years. [1]. Children who during their childhood stage have obesity or overweight, are more likely to be obese in adulthood [2]. An increase in daily physical activity (PA) levels can mitigate the effects of obesity and other non-communicable diseases derived from sedentary habits and poor nutrition [3]. Therefore, is vital promoting adequate PA behaviours during the childhood to ensure its persistence in adolescence and adulthood [4, 5], as key to preventing diseases along the life [6]. Other positive effects of an appropriate PA on youth health have been described, such as the improvement in the academic and cognitive achievement [7], a finer sleep health [8], and enhanced health-related quality of life [9]. Additionally, physical inactivity continues to increase worldwide, however, in South American countries such as Ecuador [10, 11], type 2 diabetes, hypertension, and strokes have been presented as the main causes of death [12], with a larger disease prevalence in urban areas [13].

An interesting way to encourage school-age young people to increase their PA levels is to help them take advantage of every opportunity to be active throughout the day [14]. In this sense, Sallis et al. [15] exposed a model based on four domains of active living: recreation, transport, occupation, and household, where the main influences of the built environment and social policies were identified. Thus, active daily behaviours such as active commuting to school (i.e. go to/from school by walking or cycling) is presented as an optimal option to incremnet PA levels in young school students to protect and improve their health, especially, their cardiovascular health [16, 17]. Until date, few studies about ACS have been conducted in the Latin American population beyond Brazil [18], Chile [19], or Colombia [16, 20], and more studies from other South American countries are required.

A recent study identified a range of predictors of ACS in children and adolescents based on an ecological framework, including individual, social/cultural, built and policy environment factors [21]. Young people’s age [22] and gender [23] are personal characteristics which have been shown to affect the ACS rates, where adolescents and boys were more likely to actively commute to school than children and girls respectively. However, the relationship between ACS and other individual factors such as aspects of a youth’s health still need to be better clarified. Sleep duration and breakfast consumption could be considered relevant health behaviours in both children and adolescents, due to its link with numerous chronic diseases [24–26]. Sufficient sleep duration requirements vary across the lifespan, and from person to person, but it is important to take into account that individuals who habitually sleep outside the normal sleep range may be compromising their health and well-being [27]. In the young population, the recommended range of sleep duration so as not to compromise health is from 9 to 11 and 8 to 10 h in children and adolescents respectively [28]. Recent studies have demonstrated that children and adolescents with short sleep durations had greater odds of being overweight or obese [24, 25], a higher risk of elevated fasting glucose levels [29], and an unhealthier immune profile [30]. Moreover, children who do not usually have breakfast present higher blood markers related to cardiometabolic risk [26]. These two lifestyle factors have also been associated with changes in physical activity patterns. Short sleep duration in young people has been associated with low physical activity levels [31], whereas skipping daily breakfast has been linked to sedentary behaviours such as increased screen time [32]. However, there are few reports in the literature analysing the relationship between these lifestyle factors and ACS. Thus, more attention needs to be paid to daily lifestyle factors immediately before commuting to school, since they could strongly affect ACS choice, as has been demonstrated [33].

In a study carried out by Martinez-Gómez et al. [33], Spanish adolescents with poor sleep duration on school days and habitual breakfast consumption reported the lowest percentage of ACS. On the other hand, a study conducted in Brazil exhibited that adolescents who commute actively to school reported lower sleep duration than passive adolescents [32]. This evidence suggests the need to analyze more deeply the relationship between sleep patterns, daily breakfast and ACS, and especially how they could differ between age groups and gender. The aims of the current study were: 1) to analyse the prevalence of active commuting to school, sleep duration, and breakfast consumption according to age group and gender; and 2) to analyse the association between ACS, sleep duration recommendations, and breakfast consumption according to age group and gender in a sample of Ecuadorian school-age students.

## Methods

### Study design and sample

We conducted a cross-sectional study as part of the first phase of the “PACO: **P**edalea y **A**nda al **CO**legio” Ecuador Study. The PACO Ecuador Study examined the prevalence of ACS in Ecuadorian children and adolescents. In total, 732 children and adolescents, aged 10 to 18 years old, belonging to three public urban schools in Riobamba (Ecuador) and selected by convenience sampling participated in this study. They were categorised into children (aged between 10 and 12), young adolescents (aged between 13 and 15), and older adolescents (aged between 16 and 18). This categorisation was selected because adolescence is a period with several puberty-related changes, and so the characteristics and decisions of early adolescents might be different to those of older adolescents [34]. All measurements were taken between April 2014 and May 2015. The three schools were in the same region and had similar weather conditions: the average temperature during the collection was 14 °C (https://www.wunderground.com). The school board, parents, and students were informed about the study and they agreed to participate. Students were invited to participate after the research team had visited the schools. The Medical Ethics Committee of the National University of Chimborazo (Riobamba, Ecuador) approved the study design, study protocols, and informed consent procedure (code: 46-CI-2015-07-02).

### Measurements

#### Socio-demographic characteristics

Children and adolescents self-reported their age, gender, and home address. The distance from home to school was calculated using Google Maps [35], and we used the shortest network path between each student’s home address and school, measured in metres. The children and adolescents were of low to middle socioeconomic status (SES, 1–3 defined by the Ecuadorian government).

#### Active commuting to school

Student modes of commuting to school were assessed using separate questions about travelling to and from school: “How do you usually travel to school/come back from school?”. The students were asked to select one of the following response categories: walking, cycling, car, motorcycle, bus, train, and others. Children and adolescents were categorised as “active commuters” if they reported that they usually walked or cycled to and/or from school (i.e. at least one trip, going or coming back, either on foot or by bike), and as “passive commuters” if they usually travelled to and from school by car, motorcycle, bus, or train (i.e. both trips, going and coming back, by passive transport). This questionnaire was proposed as one of the most appropriate to ask about the mode of commuting to school [36], and has been shown to be a valid measure [37]. A Spanish and English version of the questionnaire is available at http://profith.ugr.es/pages/investigacion/recursos/paco.

#### Lifestyle factors: Sleep duration and breakfast consumption

Sleep duration and breakfast consumption were self-reported by the children and adolescents. Sleep duration was referred to the time spent in bed. The children and adolescents were also asked the following questions “What time do you go to sleep every day? What time do you wake up every day?”. They self-reported the exact time when they went to sleep and when they got up on a usual school day, and we calculated sleep duration on this basis. The students were categorised as having “Adequate sleep duration” when they met *The Sleep Foundation* recommendation for health, and “Non-adequate sleep duration” when they did not meet the recommendations. According to the recommendations, the adequate range of sleep duration for children was from 9 to 11 h, and for young and old adolescents it was from 8 to 10 h [38].

Daily breakfast consumption was assessed with the following question: “From Monday to Friday during the school year, how many days do you have breakfast?”. The participants could report: “5 days”, “4 days”, “3 days”, “2 days”, “1 day”, or “I never have breakfast on school days”. Students were categorised as “skipping breakfast” (when students reported not eating breakfast or eating breakfast on fewer than 5 days) and “daily breakfast” if they reported having breakfast on all 5 days in a usual school week [33].

### Statistical analysis

Descriptive statistics (means, standard deviations, and percentages) were reported for all relevant variables. The Chi-square test was used to examine differences in the percentages of modes of commuting, sleep duration and breakfast consumption across age groups and gender. The Kruskal Wallis test was used to analyse the differences between age groups regarding the continuous variables of distance from home to school and sleep duration. Three logistic regression models were fitted to examine the association between the mode of commuting and the lifestyle factors of sleep duration and breakfast consumption, with a separate analysis for each age group. Two logistic regression models were fitted to examine the association between mode of commuting and the lifestyle factors of sleep duration and breakfast consumption separately by gender. Mode of commuting variable was included in the model as the dependent variable (reference: actively commute to school), and sleep duration and breakfast consumption were included as fixed factors (references: meet sleep duration recommendations and have daily breakfast consumption). All logistic regressions analysis were controlled by age, gender and distance from home to school (shorter path in meters by Google Maps). A multivariate logistic regression model was performed to analyse the main predictor variables of ACS, including age, gender, distance from home to school, age groups, sleep duration and breakfast consumption habits. We conducted the analyses using SPSS, IBM (v. 20.0 for Windows, Chicago, IL, USA), and the level of significance was set at *p* < 0.05.

## Results

### Prevalence of active commuting to school, sleep duration, and breakfast consumption

The descriptive data of the participants is presented in Table 1. The students were mainly young adolescents (13–15 years old) and boys (65.3%). Their mode of commuting was mainly passive (78.5%) and the mean distance from home to school was 3561.35 ± 3277.50 m. The participants slept a mean of 8.10 ± 1.50 h per night and the children had the significantly highest mean of sleep duration (8.96 ± 1.46 h). Only 27.9% of the participants met the sleep duration recommendation by age, and most had breakfast every day (67.3%).

**Table 1:** Descriptive data of the participants

	*n* = 732	*P* value
	N (%)	
Age (*n* = 718)		
Children	246 (34.3)	
Young Adolescents	310 (43.2)	
Older Adolescents	162 (22.6)	
Gender (*n* = 731)		
Boys	477 (65.3)	
Girls	254 (34.7)	
Mode of commuting to school (717)		
Active commuters to school	154 (21.5)	
Passive commuters to school	563 (78.5)	
Distance from home to school (meters) *	3561.35 (3277.50)	
Distance from home to school (meters)* by age group		
Children	3351.40 (3275.99)	0.096^a^
Young Adolescents	3413.25 (3158.43)	
Older Adolescents	4109.90 (3503.75)	
Sleep time duration*	8.10 (1.60)	
Sleep time duration by age (hours)*		
Children sleep duration	8.96 (1.46)	< 0.001^a^
Young adolescents’ sleep duration	7.96 (1.51)	
Older adolescents’ sleep duration	7.08 (1.20)	
Sleep duration recommendations^#^ (*n* = 716)		
Adequate sleep duration	200 (27.9)	
Non-adequate sleep duration	516 (72.1)	
Breakfast consumption recommendations (*n* = 730)		
Daily breakfast	491 (67.3)	
Skipping breakfast	239 (32.7)	

*Expressed by mean and standard deviation

# Recommended range of sleep time duration by age (National Sleep Foundation’s sleep time duration recommendations, 2015)

^a^Kruskal Wallis test

Missing data: age (*n* = 14), gender (n = 1), Mode of commuting to school (*n* = 15), Sleep duration (*n* = 16), breakfast consumption (n = 1)

**Fig. 1:**
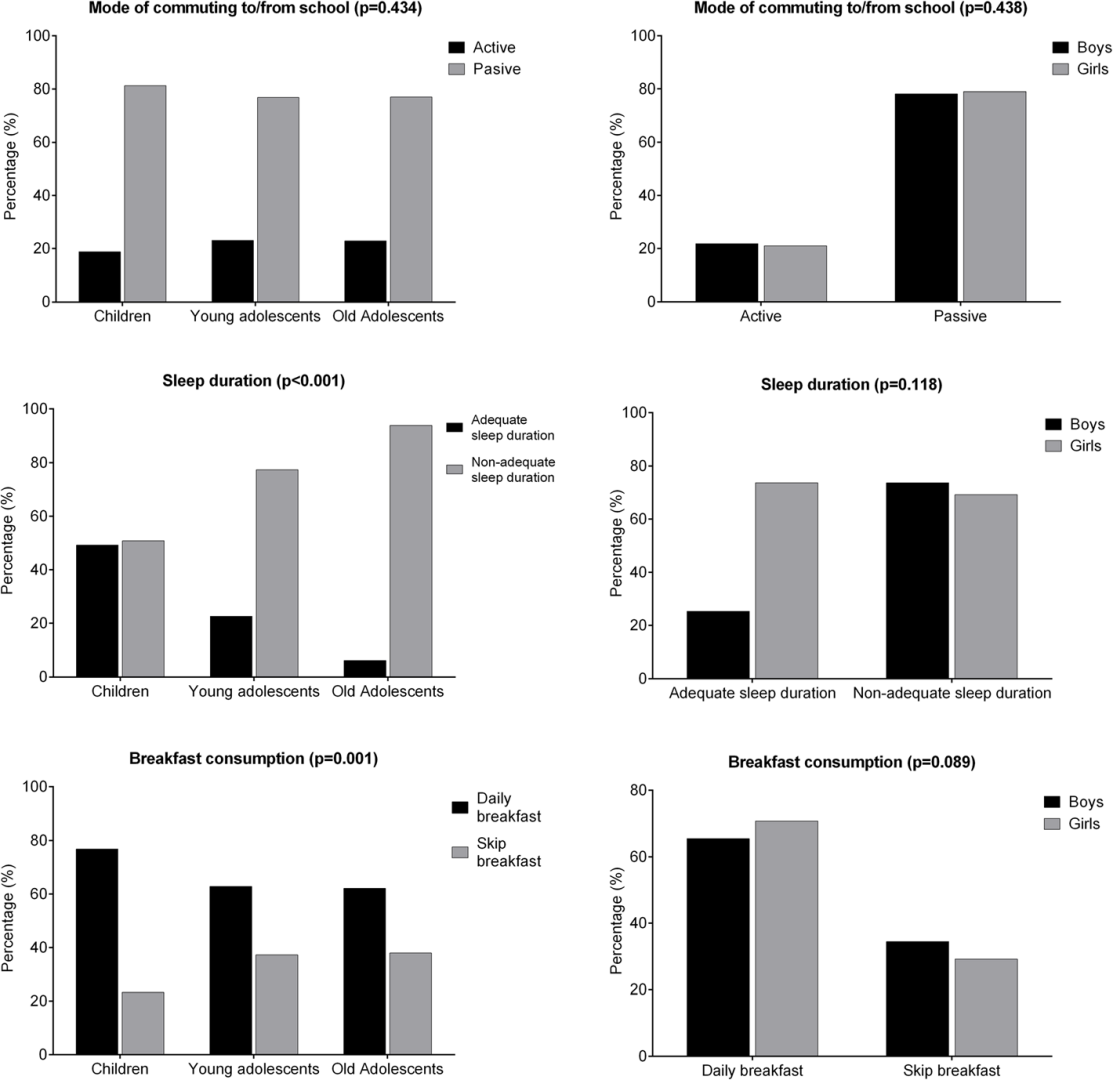
Mode of commuting, sleep duration, and breakfast consumption prevalence across age groups and gender

The percentages of the mode of commuting, sleep duration behaviour according to the recommendations, and breakfast consumption across age groups and gender are presented in Fig. 1. There were significant differences between the age groups for sleep duration and breakfast consumption. The percentage of students who met the sleep duration recommendations was lower in old adolescents (*p* < 0.001), where only 6.2% met the recommendations, and daily breakfast consumption was also lower as students grew into old adolescence (*p* < 0.001). There were no differences across age groups or gender regarding the mode of commuting, sleep duration, and breakfast consumption.

### Association between ACS, sleep duration recommendations, and breakfast consumption by age groups and gender

**Fig. 2:**
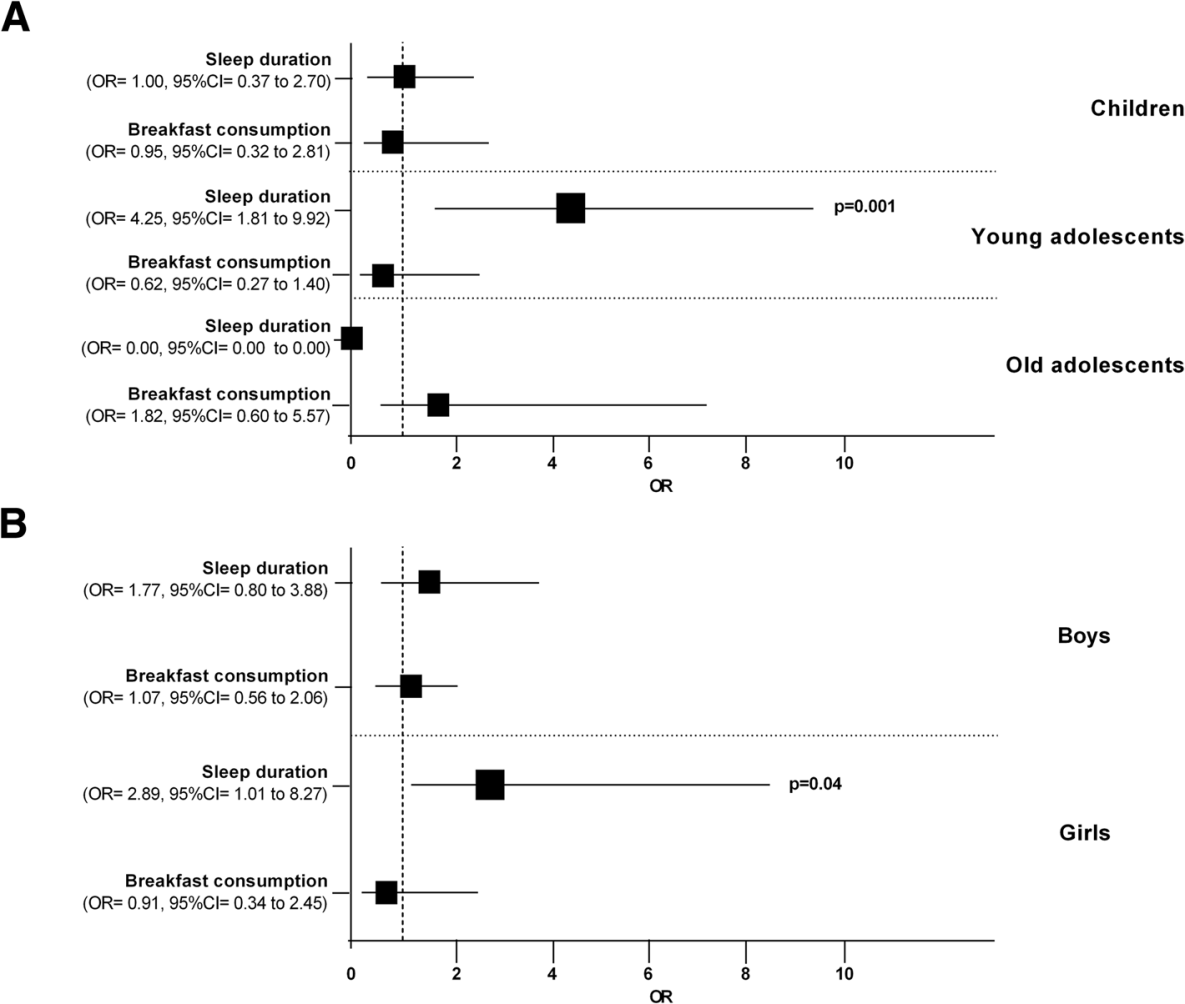
Association between ACS and sleep duration and breakfast consumption (**a**: according to age; **b**: according to gender). *ACS (ref: actively commute to school), sleep time duration (ref: adequate sleep time duration according to National Sleep Foundation); breakfast consumption (ref: having daily breakfast)

The associations between the mode of commuting (active vs. passive), sleep duration and breakfast consumption across different age and gender groups are presented in Fig. 2. There was only an association between mode of commuting and sleep duration for young adolescents. The young adolescents who met the sleep duration recommendations were more likely to be active commuters to school than their counterparts who did not (OR = 4.25; 95% CI = 1.81 to 9.92; *p* = 0.001). Consequently, there is a probability of 81% (% odds = OR/OR + 1) that young adolescents (13–15 years) who have an adequate sleep duration every night during the school week according to international recommendations, will be active commuters to school every day.

There was also a positive association between the mode of commuting to school and sleep duration according to gender. Girls who had an adequate sleep duration were more likely to be active commuters to school than boys (OR = 2.89; 95% CI = 1.01 to 8.27; *p* = 0.04). Consequently, there is a 74% probability that girls who have an adequate sleep duration every night during the school week according to international recommendations, will be active commuters to school every day. There were no more positive associations between the mode of commuting to school and the lifestyle factors analysed (recommended sleep duration and daily breakfast) in other ranges of age or according to gender.

Age (*p* = 0.007), and meet with the sleep duration recommendations (*p* = 0.013) were the independent variables significantly associated with ACS, when all independent factors were analysed in a multivariate logistic regression model (Table 2).

**Table 2:** Multivariate logistic regression model of sociodemographic and lifestyle factors associated with ACS

Variables	OR	95% CI	*p* value
Gender (reference boy)	1.02	0.59–1.78	0.922
Age (years old)	1.64	1.14–2.35	**0.007**
Age groups			
Older adolescents	Reference		
Young adolescents	5.44	0.76–38.88	0.091
Children	2.87	0.97–8.527	0.057
Distance from home to school (walking meters)	1.00	1.00–1.00	1.000
Meet sleep duration recommendations	2.19	1.17–4.10	**0.013**
Daily breakfast habit	1.02	0.59–1.76	0.945

*p* value in bold = significant association (*p* < 0.05)

## Discussion

The main result of the present study indicated that adequate sleep duration was associated with ACS in young adolescents (13-15 yr) and in school-age girls from Ecuador, however, there was no association between ACS and breakfast consumption for any of the age groups or gender. The prevalence of adequate sleep duration and daily breakfast consumption was higher in the children’s group (10-12 yr). Finally, there were no differences in the mode of commuting, sleep duration, or breakfast consumption across gender groups.

The young adolescents in this study who met with the sleep duration recommendations at night were more likely to commute actively to school. Surprisingly, there was no positive association between ACS and the children’s group, although this was the group that mostly met the sleep duration recommendations. A previous study [33] also showed that inadequate sleep duration was inversely associated with ACS in Spanish adolescents (13-17 yr). It seems that the morning fatigue caused by a lack of sleep could mediate the association between sleep duration and ACS [39], choosing inactive modes of commuting to school rather than active modes. However, according to the body of literature, the mode of commuting to school seems to be more related to social and environmental factors than to the students’ freedom of choice [32]. This could explain the lack of association between the children’s group and ACS in this study, since children are less independent than adolescents in choosing their mode of commuting to school because they are often guided by parents [40].

To the best of our knowledge, only one study analysed the association between adequate sleep duration and ACS in school-age girls with similar results [33]. In our case, the significant differences remained after controlling for gender. These differences may be due to other uncontrolled confounders (e.g. built environment data), as there is insufficient evidence available to explain the gender differences. Thus, it is important to examine in depth the association between adequate sleep duration and ACS, and especially in young girls, where a reduction in sleep hours has been observed.

There were no positive associations between ACS and breakfast consumption according to age groups or gender in our study. A previous study in Spanish adolescents showed an inverse association between ACS, and daily breakfast consumption, where adolescents who did not usually had breakfast during school days had a higher probability to perform active commuting to school [33], what does not mean that skipping breakfast must be acquired or recommended to school students to increase ACS. On the contrary, daily breakfast and ACS must be promoted as a healthy strategy for the improvement of the public health at the school setting. The authors hypothesized that starting from the idea that walking to school could lead to extra travel time, teens may prefer to spend their time having breakfast at home and not actively traveling to school, otherwise, those who travel with a faster mode of commuting but passive, may have more time to have breakfast at home before to school. These rationales need to be analysed in future research considering other key factors, such as time invested in eating breakfast, family habits, etc., since they might mediate the associations between eating breakfast and commuting to school. Finally, it is important to note that when all independent factors in this study were included in a multivariate logistic regression model, the age, and meet the sleep duration recommendations were the independent variables which significantly predicted ACS behaviour, as studies have shown previously [22, 41, 42].

In this study, the 72.1% of students did not meet the recommended sleep time during a regular school day, reaching higher percentage than the one achieved by the Brazilian adolescents aged 10–19 at 39% [43], and higher than in Argentinian adolescents aged 10–15 with a 49% [44]. However, neither of these studies analysed the differences between age groups, unlike the present study, where the older adolescents group (aged 16–18) presented the highest percentage of students with inadequate sleep duration compared with the rest of the age groups. Our findings related to gender are the opposite of two previous Portuguese studies, where girls had poor sleep duration, and in consequence, demonstrated more sleepiness at school [32, 45]. Despite this, and although we did not find significant differences in our study, the reasons for the gender difference are still unknown. It is known that the sleep regulation processes present changes throughout adolescence characterised by later sleeping and later waking up times [32]. These behaviours generate a slower accumulation of sleep, which is observed up to the end of puberty, and is related to the maturational process and its consequent gonadal development [46]. It is also worth noting that according to the Ecuador school schedule, adolescents start school before children, what added to the normal maturation process could result in less time in bed. Longer time spent on transport in long journeys to school has previously been related to a reduction in time in bed in young people [32], so that in this study, the associations between ACS and lifestyle factors were controlled by distance from home to school.

The majority of Ecuadorian school students in the current study consumed a daily breakfast (67.3%), without differences between boys and girls, and children had the highest significant percentage of daily breakfast compared to young and older adolescents (*p* = 0.001). A similar study carried out in Ecuador [11] had different results, since most of the sample (96.7%) consumed a daily breakfast, but it did not differ between gender and age groups. Similarly, a higher percentage of students who had daily breakfast was found among Spanish adolescents (86.6%), but the authors did not analyse the sample by gender and/or age groups [33]. In the present study, the age group differences could again be explained because younger students have less responsibility for making decisions about lifestyle factors such as having breakfast [47]. Despite the scarce literature, it might be assumed that healthy behaviours lead to other healthy behaviours [48]. Therefore, active commuting to school is a multifactorial behaviour and all the possible associated factors should be analysed with caution in this multifactorial approach.

The primary limitation of this study is inherent to its cross-sectional nature, and consequently, we cannot confirm whether the modes of commuting to school determine an adequate sleep duration and daily breakfast habit or vice versa. Sleep duration was also self-reported, so the results should be taken with caution. We did not perform a random sampling of the schools, so the study sample is not likely to be representative, and the data was self-reported. However, to the best of our knowledge, this is the first study analysing data about the commuting behaviours of Ecuadorian school students and their relationship to sleep duration and daily breakfast consumption, which are important daily behaviours for the health of young school students. The sample size was considerably high in relation to the demographic setting of the study. It would also be interesting to identify the number of weekly trips through active modes of transport to analyse the differences in relation to sleep and breakfast.

## Conclusion

In summary, Ecuadorian young adolescents (13-15 yr) and girls who met the recommendations for sleep duration during school days demonstrated a positive association with ACS, however, there was no association between ACS and breakfast consumption for any of the age groups or by gender. Our study suggested that Ecuadorian children (10-12 yr) were those that best meet with the adequate sleep duration and breakfast consumption recommendations. Future research could include more confounders, such as environmental factors (walkability from home to school), school schedule, or the breakfast patterns of parents, for a better comprehension of the association between ACS and healthy lifestyle factors, such as sleep duration and breakfast consumption in the young population. It is also necessary to design cohort studies to elucidate the factors associated with physical activity behaviours. Finally, the efficacy of campaigns to prevent obesity by promoting physical activity and adequate nutrition need to be tested in intervention studies on young schoolchildren in different areas of Ecuador.

## Abbreviations

ACS: Active commuting to school
PA: Physical activity
PACO: Pedalea y Anda al Colegio
